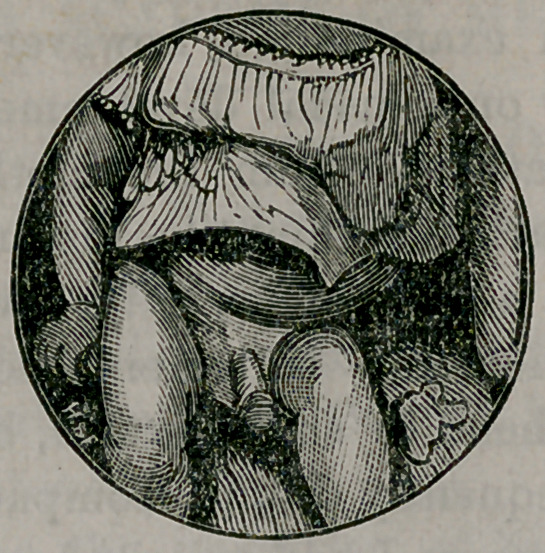# Atlanta Academy of Medicine

**Published:** 1877-11

**Authors:** 


### Reports of Societies.

### ATLANTA ACADEMY OF MEDICINE.

ATLANTA, GA., October 23, 1877.

Society met, Dr. V. H. Taliaferro, President, in the chair.

On the call for reports of cases,

Dr. W. F. Westmoreland mentioned that of a boy seven years old, from Spartanburg, S. C., who had been a sufferer for five years with stone in the bladder.

Three days since, the operation of lithotomy, for its removal, was made, and a rough stone, one by three-fourths of an inch, was extracted. The disproportion between the size of the stone and the parts of the child concerned in the operation, was such that it was with difficulty removed from the bladder without lacerating the perineum. The stone was exhibited as one of unusual size, found in the bladder of a child so small.

He also exhibited a pathological specimen from the case of necrosed maxilla reported at the meeting of 9th inst. The peculiarities of the piece of necrosed bone presented, to which he desired to call attention, were the excessive hardness of the specimen, and its resemblance to some preparation of iron, in color and general appearance.

Dr. Baird called attention to the subject of hysteria, and mentioned a case recently treated by himself with chloroform, by inhalation, in which unconsciousness and semi-coma were prominent symptoms. He found the remedy successful in rousing the patient from the unconscious state, and thinks such nervous condition may generally be promptly relieved by this means.

Dr. Todd asked whether any member had recently met with scarlatina in his practice. None answering, he reported the case of a child affected with this disease, in which, besides the usual symptoms, that of urticoria was prominent, and, for sometime, very distressing to the little subject.

The President, Dr. V. H. Taliaferro, reported a case of infantile masturbation, and said:

I was first called to see the babe some two months ago, on account of the mother's anxiety respecting the genito-urinary organs. From her previous statement to me, I expected to find some malformation, or strikingly abnormal condition of these parts. Upon examination, however, I found what appeared to be simply on unusual development of the penis and testicles. I then gathered from the mother what I conceive to be a most remarkable infantile history. Although I regarded the mother a lady of veracity, I was unwilling to accept in full her mournful statements concerning her babe, without verifying them, to some extent, by personal observation, which I subsequently did, in company with my friend, Dr. J. T. Johnson, whom I had solicited to see the case.

The history given me by the mother of the babe is as follows: Age, nine months; second child; weight, at birth, five pounds, with full winter dress; born at term; tall enough, but very thin and lean; appeared to be well, and as active and vigorous as is usual with children at birth. During pregnancy the mother had been impressed with the idea that something would be wrong with her child, and immediately inquired, at its birth, if it was all right. Upon her recovery from a serious attack of sickness of some weeks duration, she again inquired as to her child, and was told by her medical attendant (homœopath) that there was a "little something wrong." Her anxious inquiries elicited the statement that there was "a malformation." Inquiring earnestly as to the nature of the malformation, she, by her doctor, was told that there was "an unusual development of the penis, and, if he lives, will be a great rascal." The so-called malformation was made the subject of frequent remark and discussion by the nurse and lady visitors to the lying-in chamber. The result of these discussions was, that the child was destined for *a great scamp.* Passing through the usual ills of baby flesh, in the way of "hives," jaundice, thrush, etc., up to the age of five months, he began testing the powers of his genitals by what his mother termed "teasing himself." This *teasing*, at first very occasional, and usually early of mornings upon awaking, became more and more frequent until, now, its mother has become distressed and alarmed, and hence her appeal for medical advice. She states that the *teasing* was done by working the legs while he lay upon his back. She stated that these acts were attended with great excitement and evident enjoyment, and with *complete erections of the penis.*

The wood cut is made from a photograph when the child was ten monts old, at which time he had been masturbating five months, and certainly quite an adept at the business. It will be seen that the genital organs are out of all proportion to his size and age. The glans is large for the penis, the prepuce rolls back, leaving the glans completely and constantly uncovered. The testicles occupy the scrotum—are large and pendulent. For some two or three months, he suffered from teething and diarrhœa, from which he became greatly weakened and emaciated, in the meantime keeping up his habit with increasing energy and industry. With an improvement of his health, which, for the past few weeks has been decided, the acts of masturbation have been less frequent. He now is quite playful, and amuses himself with his toys, crawling over the floor, etc., and, as his mother says, "forgets to tease himself," but if, by any accident, his attention is drawn to the penis, or he ceases his plays, he at once falls on his back and commences vigorously his favorite amusement. A crying spell always ends, if left to himself, in masturbation. The frisking motion of the legs seems to call his attention to his old habit, and at once the whole expression of the face is changed from that of pain and distress to one of enjoyment.

At my solicitation, the child was brought to my office for the purpose of witnessing, if possible, the act and the manner of the masturbation.

Dr. T. J. Johnson was present by invitation to witness the strange procedure. The child was placed on his back upon a table, and at once commenced crying and looking towards his mother, as though he wished to be taken up. In a little while he ceased crying, and commenced a rather rapid motion of his legs, with the thighs flexed upon the abdomen, and the knees crossed, one over the other. The motion of the legs was not an irregular jerking, but a systematic regular movement, rather transversely to the pelvis, the knees well crossed. In a little while after commencing his movements, we noticed that he gathered, or rather spasmodically clinched, his napkin with one hand, and drew it tightly. This, he evidently had learned, to make the greatest possible amount of friction upon the penis. As he proceeded with the act he seemed to become thoroughly absorbed in its vigorous execution. With his head rigidly thrown back, the eyes rolled up, lids partially closed, mouth wide open, and jaws rigid, cheeks intensely flushed, perspiration bathing his forehead, and with each rapid and quick motion of the legs a grunt, we had presented to us a novel scene, and certainly one of considerable interest, both in a physiological and pathological view. Removing his napkin, so as to expose the penis, we found a tolerable degree of erection. His movements were continued without the napkin, apparently much to his annoyance. The mother stated that the degree of erection witnessed by us was nothing like so rigid as was usual with him when left to himself. At this time the child was in delicate health from teething—very feeble, and quite emaciated. Since, however, his health has greatly improved, and he is now quite well—is bright, cheerful and very playful. His photograph was taken very soon after the visit spoken of to my office, now some six weeks.

Have we here simply a physiological or a pathological conviction? My own opinion, from a rather careful study and observation of the case, is, that it is purely physiological, and the result of premature development of the genital organs. I will be glad if Dr. Johnson will state to the Academy his observations and impressions of this case.

I will here state, that upon careful inquiry into the family antecedents, I learned that the mother had an unusual development of the clitoris, and that an uncle was possessed of extraordinary development of the penis, which, in childhood, had given some anxiety and trouble. It was owing to these peculiarities that the youthful mother apprehended something would be wrong with her babe.

In answer to the question whether acts of masturbation were frequent, Dr. T. replied that they were repeated as often as occasion or convenience afforded—perhaps several times during the day. To the question, whether any discharge occurred during the act, he replied that the mother reported an occasional discharge of clear, tenacious fluid, supposed to be mucus.

Dr. Todd remarked that he had seen in some medical work the report of several cases of this kind. One he remembered was about ten months old. The treatment consisted of cold water and bromide of potassium. In one case the habit was discontinued on changing the nurse.

Dr. Baird thought that bromide of potassium is not the proper remedy for this habit in such precosities, as the influences that prompt the act are more physcal than mental in their character. He thought that contact of the thigh with the precociously developed penis excites the disposition, and leads to the habit in question. Bromine, acting as it does upon the brain, lessening its hyperasthesia, will thereby lessen the venerial desire in the adult. Not so in the case under consideration, where the origin is evidently mechanical. He thought such mecanical means as will prevent touching or friction will be more useful than brain tonics or sedatives.

Dr. Todd thought this strange precosity has intimate connection with the organs of the mind—that no such disposition could exist, nor such habit indulged, from mechanical cause alone.

Dr. W. F. Westmoreland agreed in the main with Dr. Baird, and thought the case reported by Dr. Taliaferro was not one of masturbation proper. In the adult the discharge of semen constitutes the habit and source of detriment. Friction, mechanical entirely in character, is evidently the cause of the practice in this case.

---

ATLANTA, February 30, 1877.

The Academy met, Dr. V. H. Taliaferro, President, in the chair.

On the call for reports of cases,

Dr. Todd alluded briefly to a case of obstruction of the bowels, to which he was called in consultation with Dr. Hart, of Cross Keys. As reported to him on arrival, the patient's bowels had not been freely evacuated in about three weeks. He was, at the time, suffering very much with pain in the abdomen, and had frequent attacks of vomiting. In the right illiac fossa there was considerable prominence, and general fullness of the whole abdomen. The abdominal enlargement seemed to depend on watery and solid accumulations, without evidence of collection of gas. He had taken castor oil and perhaps other cathartics; also an attempt at injecting fluids into the rectum and colon had been made, with poor success, owing to seeming pressure upon the pelvic viscera by the accumulations in the bowel above the obstructed portion, as was supposed, falling into this dependent position. The pulse was frequent, numbering 130.

In the consultation he advised the introduction of a large male catheter through the rectum, in order to fill the colon below the supposed stricture or impaction. It was found, however, that only about two pints of water could be introduced before such intense suffering was produced that they were forced to desist from further attempts to force more. He had previously taken morphine by the mouth in one-fourth grain doses, which was now repeated hypodermically in the same quantity. They concluded to continue the opiate occasionally, and the injection as often and in as large quantity as could be borne without too great suffering.

A letter was read from Dr. Hart, written a few days after Dr. Todd's visit, giving the gratifying intelligence, that after continuing for two days the course of treatment agreed upon, and commenced before the consultation ended, fecal discharges commenced and continued to the thorough emptying of the canal, and perfect relief of the patient.

He desired to call attention to another case, having also a fullness amounting to a tumor in, or just above, the right illiac fossa, supposed at first to depend on impaction of the cœcum and ascending colon with hardened feces. He was led to this suspicion from the number and character of scyballa discharged from the bowels after injections. Symptoms, however, were soon developed which proved that an abscess in the abdominal wall gave rise to the tumor. Finally it was opened, and discharged a large quantity of pus.

Dr. Baird desired to know where Dr. Todd supposed the watery accumulation, alluded to in the case first mentioned, was contained—whether in the cavity of the peritoneum or in the bowels? Dr. Todd answered, in the small intestines.

The President, Dr. Taliaferro, in connection with the case of obstructed bowels reported by Dr. Todd, advised the use of ox gall in obstruction from hardened or impacted fæces. He is in the habit of using it for accumulation in the rectum, in cases subjected to operations on pelvic organs, requiring the lower bowel to remain quiet, causing an accumulation of fæces in the rectum difficult to discharge. In such cases, the remedy is injected so as to come in contact with the hardened mass, and, according to his experience, readily softens it, so that it may easily pass from the bowel. He also referred to the use of melted lard, taken into the stomach, as a means of relieving obstructions to the passage of the contents, from unknown causes. He alluded to a case in which he was recently called in consultation, that recovered after this plan of treatment had been adopted, at the instance of Dr. Pinckney,

Dr. King alluded to the use of air injected into the colon for the relief of obstruction from intussuseption or stricture of any kind. He is of opinien that air may be injected readily, and will more perfectly and speedily dilate the bowel, so as to relieve any difficulty in which water has proved successful, and much more certainly.

Dr. Calhoun invited the attention of members to the unusual prevalence of hemorrhage of the retina. Whether the season or peculiar condition of the atmosphere led to it, he is not sure. He had met with several cases recently, in which the hemorrhagic disposition in this part seemed to continue longer than usual—the absorption taking place speedily after the blood was effused, and in a short time reappearing. In some instances temporary blindness resulted from the exuded blood. He is aware of the fact that lying-in women are frequently the subjects of this derangement, but it is in that case probably the result of parturition. This difficulty is, he thinks, sometimes the cause of permanent blindness in old persons, and not unfrequently terminates in apoplexy in such subjects.

Dr. Simpson reported a case of obstruction to the passage of fæces though the bowels, he supposed somewhat similar to to that mentioned by Dr. Todd. The patient, an old lady some seventy years of age, was taken on Saturday, the 30th inst., with abdominal pain and constipation. At first an opiate was given, with only temporary relief, and was followed by bismuth, soda and ipecac. The next day the same was repeated, with calomel and injections additional, which gave comfort, and was continued at intervals till Tuesday, when fever and pain came on, and, in consultation with Dr. Logan, large injections of water in the bowels, and oil of turpentine by the stomach, with opiates, were used. On Wednesday, the fifth day after the attack, there was fulness of right hypochondrium and general abdominal distention, pain and fever. No thorough evacuation was produced, and the patient's strength rapidly failing, she died late in the afternoon.

Dr. Todd, calling attention to the case of precocious masturbation, reported at the last meeting, said that since his attention had been called to the case by Dr. Taliaferro, he, in looking at the literature of the subject, came across the following in the *National Medical Journal:* "Transactions of the Medical Society of the District of Columbia. Precocious Masturbation. February 16, 1870. Dr. Thomas Miller related the following case of a child ten months old, who exhibited the precocious tendency of rubbing itself, and compressing its penis, until it produced a regular orgasm. But little attention was paid to the fact at first, as it was only noticed occasionally; but a month later the act had been repeated twice in fifteen minutes, and the orgasm lasted for at least a minute, leaving the child as relaxed as if it had been an emission, but which did not take place—with perspiration on the brow, pale lips and hurried breathing. At the commencement the penis would become perfectly erect, and at its close would relax and fall. The child passed urine in a free stream, in the Doctor's presence, and soon after commenced the excitation by pressing its abdomen and crossing its legs. On recovering, it played around, and in fifteen minutes time repeated the act, which almost amounted to a convulsion. The child was well developed, healthy and hearty, with a good wet nurse. He thought the brain might be affected, and gave grs. ij of potass., bro.

ter die, and cold ablutions to spine as often. Whenever the impulse was noticed cold water was applied to the pubes, and the child gradually lost the habit. Three or four days ago the habit was not practiced oftener than once in forty-eight hours, and it has no longer frequent erections. He did not examine the bladder or urethra, but there was an elongation of the prepuse, the bowels were open, and the appetite good. It sleeps alone on a hair mattress and pillow. Its nights were restless before taking the medicine, but the act was never committed until after the warm bath in the morning. The nurse was not allowed to indulge in stimulants. The earliest cure he had before seen was in a child of six years, with a large penis and emissions.

Dr. Antisell thought the case might be as well referred to reflex irritation of the spinal cord as of the brain.

Dr. Hall thought that the condition of the spine ought to be carefully noticed in such cases, and mentioned the case of a gentleman who, when recovering from a case of protracted sickness, and very much emaciated, while lying upon a sofa, upon his back, suffered from frequent emissions, and found that a thick welt in the pantaloons, pressing upon the spine, was the cause.

March 9.—Dr. Miller reported that the child had almost stopped the habit, now making its attempts only once in two or three days. After one of its orgasms, a *mucous discharge* had been noticed. He was at present using a stimulating liniment to the spine.

October 10, '71.—Dr. Miller states that he continued to attend the child for a year after the first report, and that the child had entirely recovered. The use of the bromide of potash and the ointment was continued for several months. A French nurse was employed by the family, who, at the Doctor's suggestion, was discharged, soon after which the habit subsided. Circumcision was proposed, but rejected by the parents.

Dr. Busey had seen a report by Vogel of masturbation in a female child of eleven months.

In the mortuary tables of the Leeds Infirmary, Dr. Fothergill, of England, reports a death from excessive tittillation of the clitoris in a child twelve months old.

ATLANTA, GA., NOV. 6, 1877.

The President, Dr. V. H. Taliaferro, in the chair.

The call for reports of cases was first answered by

Dr. E. L. Connally, who said he had recently under treatment a case of diphtheria in a child of five years old. The characteristic pseudo-membrane was distinct on both tonsils, and the usual eruption on the skin remained for about four days. The case not having alarming symptoms, no other treatment was found necessary than chlorate of potassium as a gargle and aperient cathartics.

Dr. Todd alluded to the case of scarlatina reported by him at the meeting of 23d ultimo, and stated that the further progress of the case and concluding symptoms confirmed the diagnosis. Desquamation occurred, as usual in this disease, after convalescence.

Dr. Baird reported a transverse fracture of the femur, three inches above the knee, in a lad fourteen years old. For six days the limb had been confined in splints consisting of four short boards extending the length of the thigh, and one long splint extending from the buttock, where counter extension, in some degree, was afforded, to a point beyond the foot. This was intended to keep the limb steady, while extension was kept up by a weight and pully, the foot being raised a little above a level with the body. The support to the limb thus afforded not being at all satisfactory, the plaster of Paris bandage was applied, with the effect of retaining the fractured ends in perfect apposition, and the whole limb in a comfortable condition. The position could be changed at will, without disturbing the fracture, and the patient thus saved from the discomfort of confinement to one posture. So perfect is the support afforded, that the patient might probably walk on crutches without detriment.

Dr. Wilson asked the comparative value of vanilla with that of plaster of Paris bandage in fractures.

To which Dr. Baird replied that he did not have any experience with the former, and was entirely satisfied with the support afforded by the plaster. He could conceive of great advantage over vanilla and starch, in the rapidity with which plaster of Paris dries and becomes firm.

Dr. Taliaferro remarked that an inspection of Dr. Baird's case impressed him with the advantages of plaster of Paris bandages, and that he was delighted with the firm support afforded, allowing the patient to move readily without disturbing the fracture.

Dr. Connally thought that vanilla having greater strength and durability than plaster, may therefore be preferable in some instances.

Dr. Wilson thought vanilla preferable on account of lightness and superior strength; the only disadvantage being the length of time required for drying.

Dr. Logan called attention to his treatment of dysmenorrhœa, the result of flexions, by the use of short cloth tents, as reported to the Academy on the 16th ultimo. Recent report from the patient thus treated confirms his good opinion of the plan, and encourages him in the hope of relieving others by this means.

Dr. J. G. Westmoreland reported a case of imaginary continuance of disease which had long since subsided. A young man had contracted gonnorrhœa more than a year ago, and, from the history given, was relieved in the usual time. The great anxiety and concentration of mind on the subject, however, caused every sensation of pain, or even of slight uneasiness to be magnified in the imagination to the degree of real suffering, and the sufferer considered them evidences of existing gonnorrhœa. The answers to direct questions proved that no symptom of the disease existed, and yet he was a wretched sufferer with imaginary disease, which he supposed had existed all the time. This state of mind had doubtless been induced by the earnest and constant concentration of his thoughts on the subject of his disease, about which he had, from the attack, felt great solicitude. This state of mind amounts to a species of mania, and the case may be denominated *gonorrhœal mania.* The treatment advised was that of convincing the patient that no symptom of the disease now exists; and though he cannot at once accept this as a true statement of his case, yet that he must exercise constant effort to do so, and indulge no thought on the subject of gonorrhœa. Similar cases had been presented to Dr. W. heretofore, which were found somewhat difficult to relieve from the erroneous impression made upon the mind, by intense anxiety and thought concentrated on the disgusting disease. The plan above mentioned, however, he thinks, if persisted in, will generally succeed.

Dr. Taliaferro had observed this state of mind, caused by the long continuance of chronic disease.

Adjourned.

## Figures and Tables

**Figure f1:**